# Prenatal hydrometrocolpos as an unusual finding in Fraser syndrome. Case report

**DOI:** 10.1515/crpm-2022-0038

**Published:** 2023-03-10

**Authors:** Isabella Dávila Neri, Adriana Patricia Farias Vela, Rafael Leonardo Aragón Mendoza, Roberto Gallo Roa, Giovanni Carlo Russo Vizcaino

**Affiliations:** Universidad De La Sabana, Hospital, Universitario de La Samaritana, Bogotá, Colombia; Hospital Universitario De La Samaritana, Bogotá, Colombia

**Keywords:** cryptophthalmos, Fraser syndrome, hydrometrocolpos, prenatal diagnosis, syndactyly, ultrasound

## Abstract

**Objectives:**

Fraser syndrome is a rare congenital malformation characterized by cryptophthalmos, syndactyly and urogenital tract malformations. The association with hydrometrocolpos is infrequent, with only a few cases reported in the literature.

**Case presentation:**

A 19-year-old primigravida presenting at 35 weeks of gestation, with prenatal finding of hydrometrocolpos associated with hypotelorism and microphthalmia. Pre-term cesarean delivery was performed due to breech labor and perinatal death. The autopsy confirmed hydrometrocolpos secondary to vaginal atresia and imperforate hymen, associated with cryptophthalmos, syndactyly, nasal and pinna malformations, confirming the diagnosis of Fraser syndrome.

**Conclusions:**

Fraser syndrome is usually a postnatal diagnosis. The association with genital abnormalities explains the finding of hydrometrocolpos, which could be considered a diagnostic criterion for this syndrome.

## Introduction

Fraser syndrome (FS) is a rare autosomal recessive congenital malformation of which more than 250 cases have been reported in the literature, with an incidence of 0.43 cases for every 100,000 births and 11.06 cases for every 100,000 stillbirths. Up to 24.8% cases, are associated with consanguinity among the parents, and recurrence among siblings is 25% [1, 2]. Mutations in the FRAS1 and FREM2 genes have been described as etiological factors [3].

FS expression varies significantly, prenatal diagnosis is infrequent, with postnatal diagnosis being made on the basis of neonatal physical exam findings. In 1986, Thomas described the major and minor diagnostic criteria, the former including syndactyly, cryptophthalmos, genital abnormalities and a family history of a sibling with FS, and the latter being congenital malformations of the nose, ears, larynx, cleft lip and palate, skeletal defects, umbilical hernia, renal agenesis and mental retardation. The diagnosis is confirmed with the presence of 2 major and 1 minor or one major and four minor criteria [4, 5]. Genital malformations in FS include vaginal agenesis, vaginal atresia or imperforate vagina, leading to the development of hydrometrocolpos. However, the latter is not reported in the literature as a finding on prenatal ultrasound [5].

We present the case of a 19-year-old primigravida at 35 weeks of gestation, with a fetal finding of hydrometrocolpos, preterm delivery and perinatal demise. Histopathology confirmed hydrometrocolpos secondary to vaginal atresia and imperforate hymen, associated with cryptophthalmos, syndactyly, nose and ear malformations, confirming the diagnosis of FS.

## Case presentation

A 19-year-old primigravida at 35 weeks of gestation with no medical or family history, without consanguinity, was referred for assessment of a fetal pelvic mass found on the third-trimester ultrasound. On admission, vital signs were within normal limits and prior fetal ultrasound scans were also within normality.

**Figure 1:**
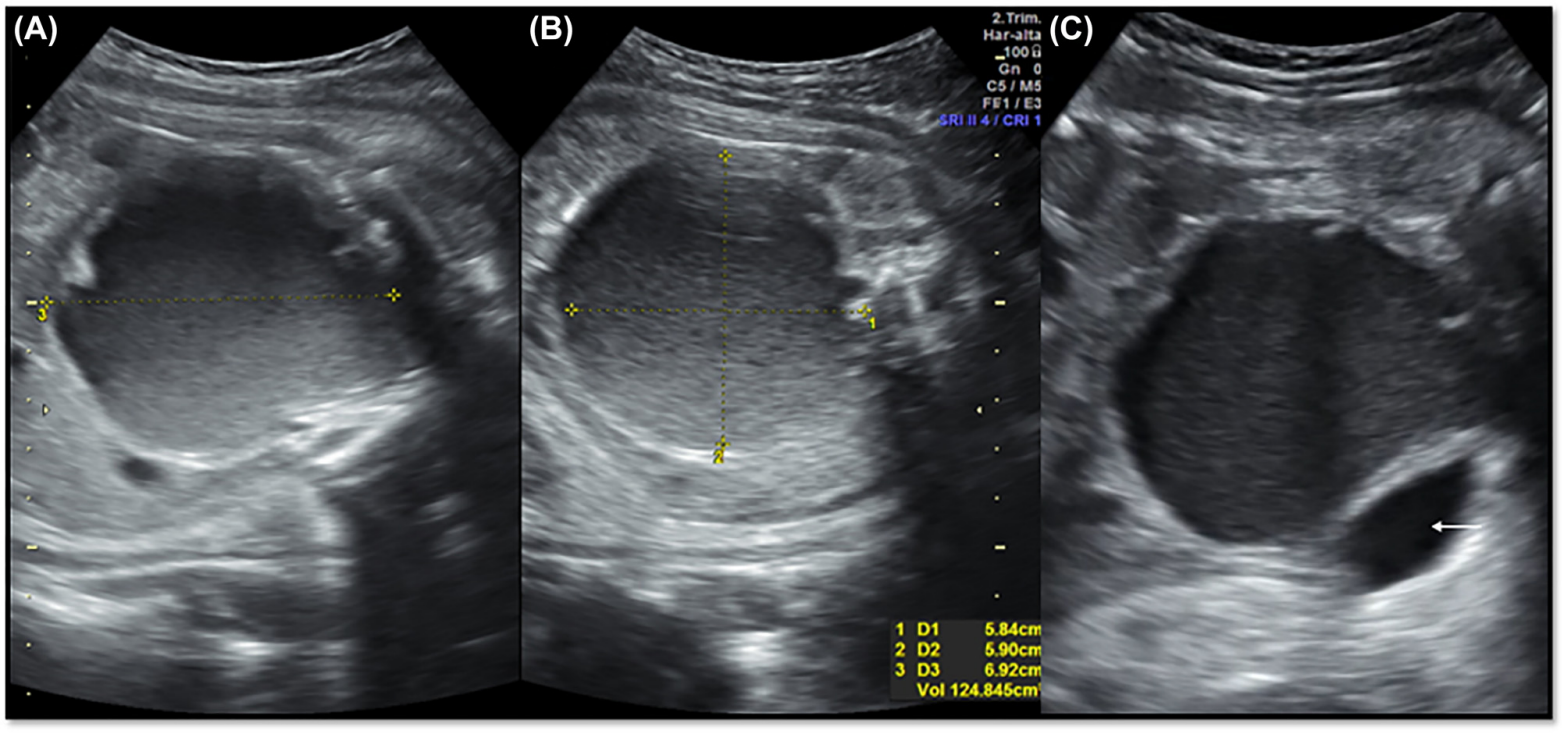
Obstetric ultrasound: (A) and (B) sagittal and axial views of the fetal abdomen with hypoechoic mass of irregular contours corresponding to hydrometrocolpos; (C) posterior relation between hydrometrocolpos and fetal bladder (arrow).

**Figure 2:**
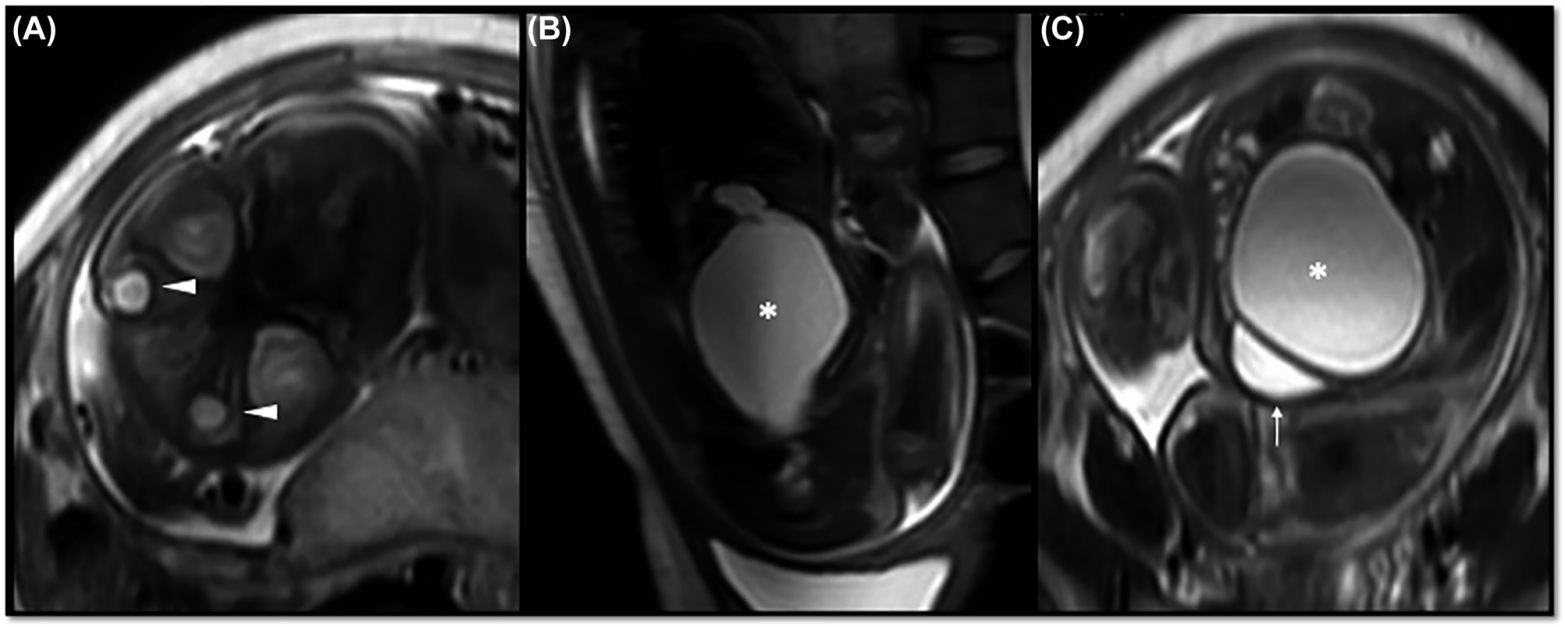
T2 fetal sequence: (A) axial section of the fetal head at the level of the orbits, showing bilateral microphthalmos (arrow heads); (B) and (C), sagittal and axial views of the fetal abdomen showing hydrometrocolpos (*) and posterior relationship with the fetal bladder (arrow).

The patient was hospitalized for workup of the fetal malformation. The transabdominal obstetric ultrasound showed a single female fetus in longitudinal position, complete breech presentation and adequate biometrics and fetal weight for gestational age. The anatomical findings included fetal head with hypotelorism and microphthalmia; fetal abdomen with a 58 × 59 × 69 mm circular hypogenic image of irregular contours and a volume of 124 cubic centimeters localized posterior to the bladder, with no uptake of color doppler; uncompromised fetal urinary system, with a diagnostic impression of fetal hydrometrocolpos (Figure 1). Complementary fetal magnetic resonance imaging confirmed the finding of hypotelorism, microphthalmia and, in the fetal pelvis, a 74 × 64 × 52 mm cystic lesion of significant size and round morphology extending from the pelvis to the abdomen, of high signal intensity in T2 and low in T1, with no septations or solid components on the inside. It caused anterior displacement of the bladder and posterior displacement of the rectum. A small saccular formation was observed in the upper portion, suggesting the presence of fluid in the uterus, consistent with the diagnostic impression of fetal hydrometrocolpos (Figure 2).

During the hospitalization, the patient had preterm uterine activity; infection was ruled out with complete blood count, C reactive protein and normal urinalysis. Late preterm lung maturation was instituted. Progression of the preterm labor and a complete breech presentation required an immediate cesarean section at 36 weeks to deliver a single fetus with normal amniotic fluid, female newborn weighing 2,550 g, APGAR of 2, 0, 0 at one, five and ten minutes, respectively. Unresponsive to neonatal resuscitation maneuvers, the outcome being perinatal death.

**Figure 3:**
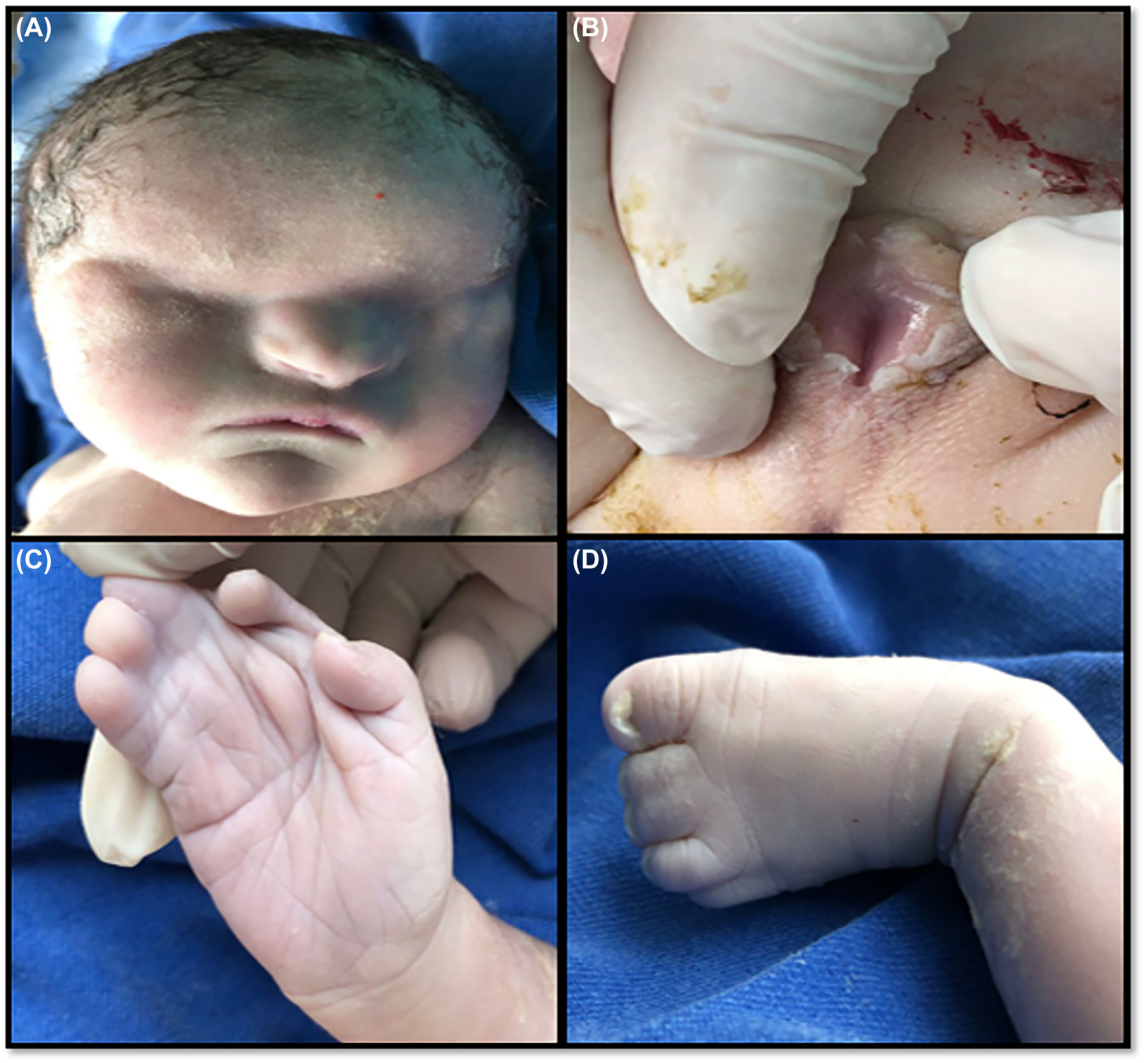
External autopsy examination of the fetus: (A) nose with broad bridge and bilateral anophthalmia; (B) external female genitalia with imperforate hymen; (C) and (D) syndactyly of upper and lower limbs.

**Figure 4:**
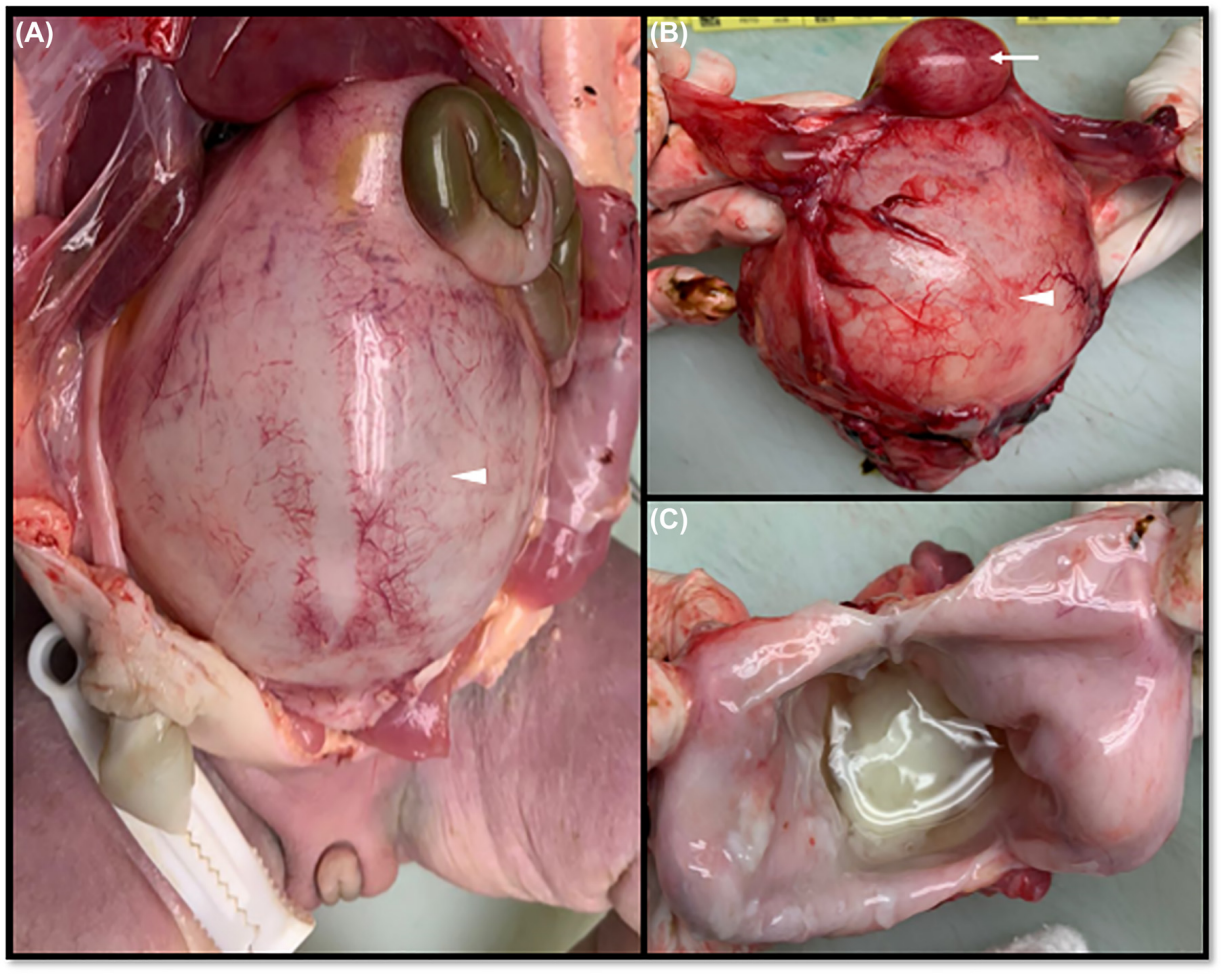
Internal autopsy examination of the fetus: (A) and (B) dilated uterus (arrow) and vagina (arrow head); (C) vaginal wall section with evidence of yellowish material inside.

Autopsy examination revealed low-set ears, micrognathia, broad nasal bridge and bilateral anophthalmia; neck and chest of normal appearance; ballooning abdomen; syndactyly in upper and lower limbs; external female genitalia with imperforate hymen (Figure 3). Internal exam of the skull and brain sections showed no evidence of gross malformations or focal lesions. In the chest, heart, lung and diaphragm were normal. In the abdomen, liver, spleen, kidneys and bladder had normal morphology. Uterus and vagina had a cystic appearance and were markedly dilated from content of yellowish material associated with vaginal atresia (Figure 4). These findings confirmed a definitive diagnosis of FS.

## Discussion

FS is an infrequent, genetically heterogenous condition caused by mutations in the FRAS1 gene in 65% of cases and in the FREM2 gene in 20% of cases. These genes encode extracellular matrix proteins essential in epidermal basement membrane and connective tissue adhesion in embryonic life; they have also been associated with defective apoptosis mechanism, giving rise to eyelid, digit, larynx and vaginal fusion [3].

It presents with a wide range of clinical manifestations, diagnosis being postnatal in the majority of cases on the basis of Thomas criteria. The most frequent associated manifestations reported in the literature are cryptophthalmos in 93% of cases, renal agenesis in 80%, syndactyly in 79%, and genital abnormalities in 66%. In our case, postnatal diagnosis was confirmed based on the presence of three major criteria, namely, cryptophthalmos, syndactyly, genital abnormality, and two minor criteria consisting of nose and pinna malformations [2, 6].

Among genital malformations associated with FS, the most frequent is clitoris hypertrophy, with rudimentary uterus, bicornuate uterus, vaginal atresia and ambiguous genitalia being less common [2, 6]. In our case, associated hydrometrocolpos was diagnosed on obstetric ultrasound, fetal magnetic resonance and postnatal autopsy, with no previous reports of prenatal found in the literature and two postnatal cases reported. The first case was a female newborn with FS, diagnosed due to the presence of cryptophthalmos, syndactyly, ambiguous genitalia and an intra-abdominal mass consistent with hydrometrocolpos [7], and the second of a 15 year-old girl complaining of primary amenorrhea, diagnosed with FS due to the presence of right cryptophthalmos, syndactyly, malformations of the pinna, nose and female genital tract with a transverse septum that required surgical management for the correction of secondary hematocolpos and hematometra [6].

Hydrometrocolpos occurs due to vaginal obstructions derived from genital tract abnormalities. This leads to the accumulation of vaginal secretions caused by hormonal influx, creating vaginal and uterine distension [8]. It is a secondary manifestation of non-syndromic abnormalities such as imperforate hymen, vaginal atresia, persistent urogenital sinus, cloacal malformations, or syndromic abnormalities like those present in McKusiCk–Kaufman, Ellis van Creveld, Bardet–Biedl syndromes [8]. Obstetric ultrasound is the main imaging tool for the diagnosis of hydrometrocolpos [9], with the usual feature being a cystic anechoic mass exhibiting air-fluid levels, localized to the midline, posterior to the bladder and anterior to the rectum [8, 10]. Fetal magnetic resonance imaging can be used as a complementary scan to guide the etiologic study of this finding, with definitive diagnosis made on the basis of the clinical findings after birth [10]. In FS cases where it is associated with genital abnormalities, these constitute a major criterion for diagnosis and may result in vaginal obstruction in female fetuses and explain the associated finding of hydrometrocolpos which, despite being infrequent, could be considered an associated diagnostic criterion.

Prognosis for fetuses with FS is usually bad and survival varies according to the severity of the associated defects. Miscarriage or preterm labor occur in 6% of cases; surviving newborns have intellectual impairment and severe mental retardation, mortality being as high as 20% in the first week of life [5]. Given the genetic association, genetics counseling should be encouraged since diagnosis and for future pregnancies, especially if there is consanguinity [3].

## Conclusions

FS is usually diagnosed after birth on the basis of the criteria defined by Thomas. Association with genital abnormalities explains the finding of hydrometrocolpos, which could be considered a diagnostic criterion, and the syndrome should be suspected in the event it is found on prenatal ultrasound.
